# Pharmacological targeting of the ephrin receptor kinase signalling by GLPG1790 in vitro and in vivo reverts oncophenotype, induces myogenic differentiation and radiosensitizes embryonal rhabdomyosarcoma cells

**DOI:** 10.1186/s13045-017-0530-z

**Published:** 2017-10-06

**Authors:** Francesca Megiorni, Giovanni Luca Gravina, Simona Camero, Simona Ceccarelli, Andrea Del Fattore, Vincenzo Desiderio, Federica Papaccio, Heather P. McDowell, Rajeev Shukla, Antonio Pizzuti, Filip Beirinckx, Philippe Pujuguet, Laurent Saniere, Ellen Van der Aar, Roberto Maggio, Francesca De Felice, Cinzia Marchese, Carlo Dominici, Vincenzo Tombolini, Claudio Festuccia, Francesco Marampon

**Affiliations:** 1 Department of Paediatrics and Infantile Neuropsychiatry, “Sapienza” University of Rome, Rome, Italy; 2 Department of Biotechnological and Applied Clinical Sciences, Division of Radiation Oncology, University of L’Aquila, L’Aquila, Italy; 3 Department of Experimental Medicine, “Sapienza” University of Rome, Rome, Italy; 4 Multi-Factorial Disease and Complex Phenotype Research Area, Bambino Gesu Children’s Hospital, IRCCS, Rome, Italy; 5 Department of Experimental Medicine, Section of Biotechnology and Medical Histology and Embriology, Second University of Naples, Naples, Italy; 6 Division of Medical Oncology, Department of Clinical and Experimental Medicine and Surgery “F. Magrassi A. Lanzara”, Second University of Naples, Naples, Italy; 7 Department of Oncology, Alder Hey Children’s NHS Foundation Trust, Liverpool, UK; 8 Department of Perinatal and Paediatric Pathology, Alder Hey Children’s NHS Foundation Trust, Liverpool, UK; 9 Galapagos NV, Industriepark Mechelen Noord, General De Wittelaan L11 A3, 2880 Mechelen, Belgium; 10 Galapagos France, 102 avenue Gaston Roussel, 93230 Romainville, France; 11 Department of Biotechnological and Applied Clinical Sciences, Division of Pharmacology, University of L’Aquila, L’Aquila, Italy; 12 Department of Radiological, Oncological and Pathological Sciences, “Sapienza” University of Rome, Rome, Italy

**Keywords:** Rhabdomyosarcoma, GLPG1790, Ephrin, Cancer stem cell, Radiation therapy, Tumour xenografts

## Abstract

**Background:**

EPH (erythropoietin-producing hepatocellular) receptors are clinically relevant targets in several malignancies. This report describes the effects of GLPG1790, a new potent pan-EPH inhibitor, in human embryonal rhabdomyosarcoma (ERMS) cell lines.

**Methods:**

EPH-A2 and Ephrin-A1 mRNA expression was quantified by real-time PCR in 14 ERMS tumour samples and in normal skeletal muscle (NSM). GLPG1790 effects were tested in RD and TE671 cell lines, two in vitro models of ERMS, by performing flow cytometry analysis, Western blotting and immunofluorescence experiments. RNA interfering experiments were performed to assess the role of specific EPH receptors. Radiations were delivered using an x-6 MV photon linear accelerator. GLPG1790 (30 mg/kg) in vivo activity alone or in combination with irradiation (2 Gy) was determined in murine xenografts.

**Results:**

Our study showed, for the first time, a significant upregulation of EPH-A2 receptor and Ephrin-A1 ligand in ERMS primary biopsies in comparison to NSM. GLPG1790 in vitro induced G1-growth arrest as demonstrated by Rb, Cyclin A and Cyclin B1 decrease, as well as by p21 and p27 increment. GLPG1790 reduced migratory capacity and clonogenic potential of ERMS cells, prevented rhabdosphere formation and downregulated CD133, CXCR4 and Nanog stem cell markers. Drug treatment committed ERMS cells towards skeletal muscle differentiation by inducing a myogenic-like phenotype and increasing MYOD1, Myogenin and MyHC levels. Furthermore, GLPG1790 significantly radiosensitized ERMS cells by impairing the DNA double-strand break repair pathway. Silencing of both EPH-A2 and EPH-B2, two receptors preferentially targeted by GLPG1790, closely matched the effects of the EPH pharmacological inhibition. GLPG1790 and radiation combined treatments reduced tumour mass by 83% in mouse TE671 xenografts.

**Conclusions:**

Taken together, our data suggest that altered EPH signalling plays a key role in ERMS development and that its pharmacological inhibition might represent a potential therapeutic strategy to impair stemness and to rescue myogenic program in ERMS cells.

## Background

Rhabdomyosarcoma (RMS), the most common soft tissue sarcoma in childhood and adolescence, arises from undifferentiated mesenchymal cells with developing skeletal muscle features [1]. In this age range, RMS includes two main histological varieties, the embryonal (ERMS) and the alveolar (ARMS) subtypes, characterized by specific genetic alterations [2–4]. Despite refinements in dose and schedule of multimodality treatment, the 5-year overall survival of patients with high-risk RMS remains < 30%, thereby requiring the identification of novel targets for a more effective therapeutic intervention [5].

EPH (erythropoietin-producing hepatocellular) proteins are a large family of receptor tyrosine kinases (RTKs), comprehending EPH-A and EPH-B receptors, which respectively bind the cell/surface-associated protein ligands, Ephrin-A and Ephrin-B [6–8]. EPH/Ephrin signalling represents a complex network of cell communications. “Forward” signalling, corresponding to the prototypical RTK mode of signal transduction, triggers the activation of signal transduction effectors, such as Rho, Ras family GTPases and AKT/mTORC1. “Reverse” signalling transduces through the Src family kinase activation [8, 9]. EPH/Ephrin network plays a key role in a wide array of developmental processes, such as cardiovascular and skeletal development, axon guidance and tissue patterning [10]. EPH-A/Ephrin-A signalling has been shown to be essential in inducing myogenic differentiation of myoblasts through the suppression of the Ras/ERK1/2 cascade [11]. Due to their physiological importance, alterations in EPH/Ephrin cascade play a central role in the pathogenesis of several diseases [12], such as cancer [13, 14], in which EPH expression levels are frequently upregulated [13–15], with EPH-A2 and EPH-B4 being the most widely over-expressed EPH receptors [13]. EPH/Ephrin role in cancer is complex by interfering in tumour initiation, progression, neovascularization, invasion and metastatization [16]. Furthermore, increasing evidence also indicates that EPH/Ephrin signalling is involved in cancer stem cell self-renewal, facilitating tumour heterogeneity, metastasis and therapeutic resistance [17]. The role of EPH/Ephrin signalling in RMS cells is still largely unknown. Although a global upregulation of EPH-B receptors and Ephrin-B ligands was previously found in RMS tumours [18], the expression levels of EPH-A receptors and Ephrin-A ligand as well as the effects of EPH/Ephrin inhibition in RMS cell biology remain unclear.

Recent efforts to design small molecule inhibitors of EPHs have generated encouraging preclinical and clinical results [19, 20]. This study describes the in vitro activity of GLPG1790, a novel small molecule that is able to inhibit various EPH receptor kinases [21]. Indeed, EPH-A2 levels were found to be significantly upregulated in ERMS tumours and cell lines in comparison with normal skeletal muscle (NSM). Treatment of ERMS cell lines with GLPG1790 in vitro significantly inhibited tumour cell growth, reduced the stem-like cell population and promoted myogenic differentiation of surviving cells. Furthermore, GLPG1790 was able to radiosensitize ERMS cells by affecting the radiation-induced activation of the DNA-repair mechanisms. At molecular levels, GLPG1790 inhibited MAPK and AKT signal transduction pathways, two well-established major drivers of ERMS oncogenicity [22–25]. Similar effects were obtained by silencing EPH-A2 and/or EPH-B2, the principal EPH receptors targeted by GPLG1790 [21], this suggesting the specificity of GLPG1790 activity. These findings indicate that EPH/Ephrin signalling is associated with ERMS development and aggressiveness, and suggest that EPH/Ephrin targeting might be a promising tool in ERMS treatment, as already suggested for other cancer types.

## Methods

### Patient clinical features

Fourteen ERMS primary tumour samples were obtained at diagnosis before any treatment from children admitted to the Department of Oncology at the Alder Hey Children’s NHS Foundation Trust, Liverpool. Histopathological diagnosis was confirmed using immunohistochemistry. Details of the patients are reported in Table 1.

**Table 1:** Clinicopathological features of the analysed tumour cases. Variables were categorized as follows: age at diagnosis (months), gender, histological subtype, primary site and clinical stage

Case	Age (months)	Gender	Histology	Primary site	Stage
ERMS1	63	Male	Embryonal	Abdominal	3
ERMS2	122	Male	Embryonal	Pelvis	3
ERMS3	61	Male	Embryonal	Bladder	3
ERMS4	45	Male	Embryonal	Trunk	3
ERMS12	18	Male	Embryonal	Bladder	3
ERMS13	40	Female	Embryonal	Retroperitoneum	2
ERMS20	60	Female	Embryonal	Pelvis	2
ERMS21	3	Male	Embryonal	Abdominal	3
ERMS23	37	Female	Embryonal	Vagina	2
ERMS27	22	Female	Embryonal	Calf	1
ERMS30	176	Male	Embryonal	Lower limb	4
ERMS43	17	Male	Embryonal	Bladder	2
ERMS44	39	Male	Embryonal	Paratesticular	1
ERMS50	22	Male	Embryonal	Infratemporal fossa	2

Institutional written informed consent was obtained from the patient’s parents or legal guardians. Control RNA was extracted from normal skeletal muscle (NSM) obtained from eight children undergoing surgery for non-oncological conditions. The study underwent ethical review and approval according to the local institutional guidelines (Alder Hey Children’s NHS Foundation Trust Ethics Committee, approval number 09/H1002/88).

### RNA isolation and quantitative real-time PCR

Total RNA was isolated by tumour tissues ground under liquid nitrogen using 1 ml of TRIzol LS reagent (Invitrogen, Carlsbad, CA) per 50–100 mg of sample according to the manufacturer’s protocol. RNA concentration and purity were measured by NanoDrop 2000 (Thermo Fisher Scientific, Inc., Waltham, MA). Total RNA (2 μg) was subjected to reverse transcription with High Capacity cDNA Reverse Transcription kit (Applied Biosystems, Foster City, CA) according to the manufacturer’s instructions. Quantitative real-time PCR (Q-PCR) for human EPH-A2 (Hs00171656) and Ephrin-A1 (Hs00358886) mRNAs was performed using the specific TaqMan Real Time Gene Expression Assays (Applied Biosystems). Each sample was run in triplicate, in at least two independent experiments, on a StepOne Real Time System (Applied Biosystems) machine. Transcript levels were normalized to the glyceraldehyde-3-phosphate dehydrogenase (GAPDH) mRNA, used as endogenous control. Relative quantification (RQ) of each mRNA in tumour samples in comparison to NSM was calculated by the comparative Ct method (2^-ΔΔCt^), using the StepOne v2.3 software (Applied Biosystems). RQ_max_ and RQ_min_, which are the maximum and minimum limits of the RQ values based on the standard error of the mean ΔCt values at 95% confidence interval, were used to plot error bars.

### Cell lines and tumour sphere culture, pharmacological and radiation treatment

The human ERMS RD and TE671 cell lines were respectively obtained by American Type Culture Collection (Manassas, VA) and Interlab Cell Line Collection (Genoa, Italy). Cell lines were maintained in high-glucose Dulbecco’s modified Eagle’s medium supplemented with 10% foetal bovine serum (FBS), 1% *v*/*v* l-glutamine, 100 μg/ml streptomycin and 100 U/ml penicillin and grown at 37 °C in a humidified atmosphere of 5% CO_2_. DNA profiling using the GenePrint 10 System (Promega Corporation, Madison, WI) was carried out to authenticate cell cultures, comparing the DNA profile of our cell cultures with those found in GenBank. Sphere-forming cells were obtained and radiation treatment performed as already described [22]. EPH receptor inhibitor GLPG1790 was provided by Galapagos NV [21], dissolved in DMSO and stored at − 20 °C until use. Chemical structural data for GLPG1790 will be published shortly.

### Cell viability, proliferation assay and FACS analysis

Cells were seeded at a density of 10^5^ cells/ml. After attachment, cells were treated with the indicated doses of GLPG1790 or DMSO for the indicated times. Cellular viability was measured using the trypan-blue exclusion assay (Life Technologies, Grand Island, NY) and the Countess II Automated Cell Counter (ThermoFisher Scientific, Waltham, MA). Data are expressed as average of triplicate ± standard deviation (SD). FACS analysis was performed as previously described [26]. Data were analysed using ModFit 3.1 software (BD Biosciences). Results were plotted as means ± SD of three separate experiments, having three determinations per assay for each experimental condition.

### Transient transfection

RD and TE671 cells were seeded at 4 × 10^5^ cells/well in 6-well plates; small interfering RNA (siRNA) against human EPH-A2 (EPH-A2^siRNA^, sc-29304 by Santa Cruz Biotechnology, Dallas, TX) and/or EPH-A2 (EPH-B2^siRNA^, sc-39949 by Santa Cruz Biotechnology) or siRNA negative control (CTR^siRNA^, sc-37007 by Santa Cruz Biotechnology) were combined with RNAiMAX (Invitrogen) and used at 60 nM final concentration; EPH-A2^siRNA^ and EPH-B2^siRNA^ are a pool of three target specific 19–25 nt siRNAs designed to specifically knock down the targeted genes. All the experiments were performed in proliferating growth medium, i.e. supplemented with 10% FBS.

### Morphological assessment

An Axio Vert.A1 microscope (Carl Zeiss Microscopy, Thornwood, NY), furnished with an AxioCam MRc5 camera (Carl Zeiss Microscopy), was used to observe the morphological changes of the cells treated with GLPG1790. Cells were photographed at 3 days post-treatment using a ×20 magnification. Images were analysed by using the ImageJ 1.47v software (http://imagej.nih.gov/ij/).

### Anchorage-dependent or anchorage-independent colony formation and wound healing assays

For anchorage-dependent colony formation assays, 4 × 10^3^ cells were plated in 6-well plates, treated or not with GLPG1790 and cultured for 8 days. Colonies were fixed in 100% methanol, stained with 1% crystal violet, and photographed. To quantify colonies, crystal violet was dissolved in 30% acetic acid in water for 15 min at room temperature (rt) and absorbance was measured using the Biochrom Libra S22 UV/VIS spectrophotometer (Biochrom, Berlin, Germany) at wavelength of 595 nm; 30% acetic acid in water was used as the blank. Colony formation capacity in GLPG1790 treated in comparison to untreated cells was calculated as follow: (OD_untreated_ − OD_GLPG1790_ / OD_untreated_) × 100%, having three determinations per experiment. For colony forming in soft agar assays, 2 × 10^4^ cells were resuspended in 4 ml of 0.33% special Noble agar (Difco, Detroit, MI) and plated (6-well plate) in growth medium-containing 0.5% soft agar. Colonies were photographed 14 days after plating.

For wound healing assays, RD and TE671 cells were plated in 6-well plates and incubated with or without GLPG1790 for 24 h. On the following day, a sterile pipette tip was used to scratch the cell monolayer (4–5 parallel scratches/plate). Cells were washed with PBS, photographed to mark scratched tracks, and incubated for 24 additional hours to evaluate cell migration into the injured areas. Wound healing was quantified using ImageJ 1.47v software. Experiments were carried out in triplicate.

### Western blot and immunofluorescence assays

Total extracts were prepared using RIPA buffer, and protein concentration was determined using the BCA protein assay kit (ThermoFisher Scientific). Western blot analyses were performed as already described [27] by using the following primary antibodies: p21 (C-19), p27 (F-8), Cyclin A (BF683), Cyclin D1 (M-20), Cyclin B1 (H-20), Cyclin E (HE12), c-Myc (9E10), Rb (C-15), integrin β1 (M-106), integrin β5 (H-96), phosphor-Src (H-3), Src (5D10C4), DNMT3B (G-9), phospho-ERK1/2 (E-4), ERK1/2 (C-14, positive also for ERK1), p38 (H-147), phospho-JNK (G7), JNK (D2), phospho-AKT1/2/3-R (Ser473), AKT1/2/3 (H-136), phospho-mTOR (Ser2448), mTOR (H-266), Myogenin (F5D), phospho-ATM (10H11.E12, Ser1981), ATM (H-248), DNA-PKcs (E-6), H2AX (C-20), Nanog (H-155), EPH-A2 (C-3), EPH-B2 (2D12C6), Ephrin-A1 (A-5), Ephrin-B2 (C-20), Ki-67 (Ki-67) and α-Tubulin (TU-02) by Santa Cruz Biotechnology; MYOD1 (MAB3878) and MyHC (05–716) by EMD Millipore Corporation (Billerica, MA); phospho-p38 (Thr180/Tyr182)(9211), phospho-H2AX (Ser139) (2577), and phospho-EPH-A2 (Ser897) (D9A1) by Cell Signaling Technology (Danvers, MA); EPH-B (total EPH-B proteins), phospho-EPH-B (Tyr317), phospho-DNA-PKcs (Thr2609) (10B1), integrin αV (P2W7) and integrin β3 (PM6/13) by AbCam (Cambridge, UK). Appropriate horseradish peroxidase (HRP)-conjugated secondary antibodies (Santa Cruz Biotechnology) were used for 1 h at rt. Protein signals were detected using Western Bright ECL kit (Advansta, Menlo Park, CA) according to the manufacturer’s instructions and visualized by ChemiDoc XRS+ (Bio-Rad, Hercules, CA). Densitometry was performed to quantify changes in protein expression using the Image Lab5.1 software (Bio-Rad). Expression and cellular localization of MyHC, MYOD1, CycD1 and p21 were assessed by immunofluorescence experiments as previously described [28]. Experiments were replicated twice.

### In vivo experiments, GLPG1790 and radiation treatment

Female CD1-nu/nu mice, at 6 weeks of age, were purchased from Charles River (Milan, Italy) and maintained under the guidelines established by our institution (University of L’Aquila, Medical School and Science and Technology School Board Regulations, complying with the Italian government regulation n.116 January 27, 1992 for the use of laboratory animals). Before any invasive manipulation, mice were anesthetized with a mixture of ketamine (25 mg/ml) and xylazine (5 mg/ml). For xenotransplants, exponentially growing TE671 cells were detached by trypsin-EDTA, washed twice in PBS and resuspended in saline solution at cell densities of 1 × 10^6^/200 μl. Xenotransplants were established by s.c. injecting in the leg of 45-day-old female nude CD1 mice 1 × 10^7^ tumour cells [23]. Treatments started when tumours reached a volume of 0.5–1 cm^3^.

GLPG1790 solution was prepared in 0.5% methylcellulose. The GLPG1790 dose used in the study had already been tested to be non-toxic to mice but effective in inducing inhibition of Ephrin signalling. GLPG170 was administered every day for 2 weeks and always before RT. Mice were irradiated using an Elekta 6-MV photon linear accelerator. Six fractions of 2 Gy were delivered at alternative days for a total dose of 12 Gy. A dose rate of 1.5 Gy/min was used with a source-to-surface distance (SSD) of 100 cm. Prior to irradiation, mice were anesthetized and were protected from off-target radiation by a 3-mm lead shield. Before tumour inoculation, mice were randomly assigned to four experimental groups. Each group was composed of 10 mice. One control group received 0.5% methylcellulose by oral gavage; one group received GLPG1790 solution at the dose of 30 mg/kg; one group received RT (six fractions of 2 Gy delivered three times/week to a total dose of 12 Gy); one group received GLPG1790 solution at the dose of 30 mg/kg and RT (six fractions of 2 Gy delivered three times/week to a total dose of 12 Gy) delivered 24 h after GLPG1790 treatment. Experiments were stopped 12 days after the last irradiation, and mice were sacrificed by carbon dioxide inhalation. Tumours were directly frozen in liquid nitrogen for protein analysis and biochemical evaluation. The effects of different treatments on tumour growth were evaluated as follows: (1) by measuring tumour volume during and at the end of experiment (tumour volume was assessed every 4 days measurement with a Vernier calliper (length × width); the volume of the tumour was expressed in cubic millimetre according to the formulation 4/3π r^3^; (2) by measuring tumour weight at the end of the experiments; and (3) by determining the time to progression (TTP), tumour progression (TP) defined as an increase of greater than 100% of tumour volume respect to the baseline. Tumour pieces were homogenized by Precellys tissue homogenizer (Bertin Instruments, Montigny-le-Bretonneux, F) for grinding samples prior to protein analysis.

### Statistical analysis

Data were expressed as mean ± standard deviation (SD) of each condition. Statistical significance between groups was assessed by Student’s *t* test, and probability (*p*) values of less than 0.05 were accepted as significant.

Continuous variables were summarized as mean and SD or as median and 95% CI for the median. For continuous variables, statistical comparisons between control and treated groups were established by carrying out the Kruskal-Wallis Tests (a parametric one-way analysis of variance for independent groups) or the Mann-Whitney test (in the case of two independent groups). Dichotomous variables were summarized by absolute and/or relative frequencies. For dichotomous variables, statistical comparisons between control and treated groups were established by carrying out the exact Fisher’s test. For multiple comparisons, the level of significance was corrected by multiplying the *p* value by the number of comparisons performed (*n*) according to Bonferroni correction. TTP was analysed by Kaplan-Meier curves and Gehan’s generalized Wilcoxon test. When more than two survival curves were compared, the Logrank test 10 for trend was used. The non-parametric rank correlation analysis between EPH-A2 or Ephrin-A1 mRNA levels and sex or disease stage was performed by determining the Spearman’s and Kendall rank correlation coefficients. To assess the probability that there is a trend in survival scores across the groups, *p* values < 0.05 were considered statistically significant. All tests were two-sided and were determined by Monte Carlo significance. The effects of the treatments were examined as previously described by Prewett et al. [29]. The effect on tumour growth was measured by taking the mean tumour volume on day 24 for the different treatment groups: controls, treatment with RT (treatment a), treatment with GLPG1790 (treatment b) and treatment with RT + GLPG1790 (treatment a + b). For tumour volume assessment, fractional tumour volume (FTV) for each treatment group was calculated as the ratio between the mean tumour volumes of treated and untreated tumours. For tumour progression, fractional TTP (FTTP) for each treatment group was calculated as the ratio between the median TTP of untreated and treated tumours. This was done for treatment a, for treatment b and for treatment a + b. The expected FTV or FTTP for the << a + b >> combination was defined as FTVa observed X FTVb observed or as FTTPa-observed X FTTP observed. The ratio FTV a + b expected/ FTV a + b observed or FTTP a + b expected/FTTP a + b observed was the combination index (CI). If CI > 1, there are supra-additive effects and if CI < 1 infra-additive ones. Strictly additive effects were observed if CI = 1. All statistical analyses were performed using the SPSS® statistical analysis software package, version 10.0.

## Results

### EPH-A2 and EPH-B signalling status in ERMS tumours and cell lines

**Fig. 1:**
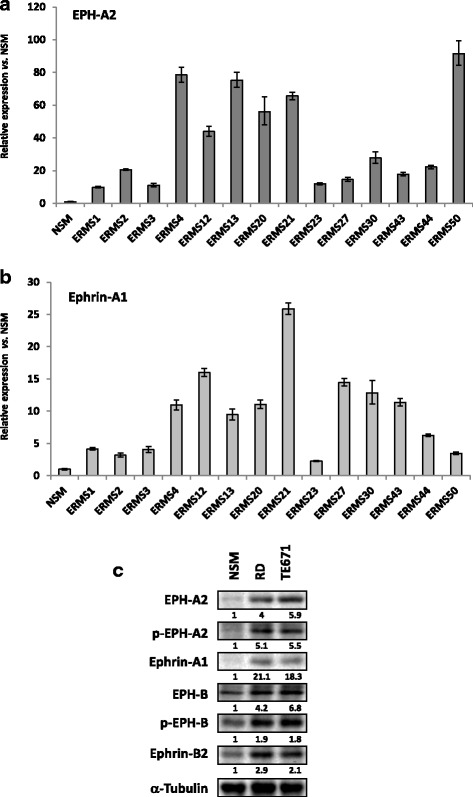
Expression and activation status of EPH-A2, EPH-B and related Ephrin ligands in ERMS tumours and cell lines. **a** EPH-A2 and **b** Ephrin-A1 transcript levels in 14 ERMS primary tumours and NSM, as measured by Q-PCR assays. GAPDH mRNA was used as an endogenous control. The relative mRNA expression levels are presented as the average fold changes (RQ) in tumour biopsies vs. NSM, set at 1. Error bars represent the RQ_max_ and RQ_min_ values of at least two independent assays, each performed in triplicate. **c** Western blots showing the expression levels of EPH-A2, EPH-B, Ephrin-A1 and Ephrin-B2 proteins as well as the phosphorylation status of EPH-A2 (p-EPH-A2) and EPH-B (p-EPH-B) in RD and TE671 cell lines in comparison to NSM; α-Tubulin was used as loading control. Representative images of three different experiments

EPH-A2 and EPH-B have been shown to be the EPH receptors most widely overexpressed in cancer [13]. Upregulation of EPH-B receptors and Ephrin-B-related ligands has been found in RMS cells [18], whilst no data have yet been reported for EPH-A2- and Ephrin-A1-related ligand. The analysis of EPH-A2 and Ephrin-A1 transcript levels, performed in 14 ERMS primary tumours by using Real Time PCR, showed that both transcripts were significantly upregulated in all tumour samples in comparison to NSM (Fig. 1a, b). No statistically significant correlations were found between EPH-A2 or Ephrin-A1 mRNA levels and gender or disease stage (EPH-A2 vs. gender: K-Tau = 0.0331, *p* = 0.9342, CI = − 0.439 to 0.516; EPH-A2 vs. stage: *r* = −0.0164, *p* = 0.9555, CI − 0.542 to 0.519; Ephrin-A1 vs. gender: K-Tau = 0.0341, *p* = 0.9323, CI = − 0.471 to 0.472; Ephrin-A1 vs. stage: *r* = 0.164, *p* = 0.5748, CI − 0.401 to 0.639. Western blot experiments revealed that protein expression levels and/or phosphorylation status of EPH-A2, EPH-B, Ephrin-A1 (EPH-A2-related ligand) and Ephrin-B2 (EPH-B4-related ligand) were significantly increased (*p* < 0.01) in ERMS cell lines in comparison to NSM (Fig. 1c). Altogether, these results and the previously reported data [18] indicate that EPH/Ephrin signalling is upregulated in ERMS.

### GLPG1790 affects viability of ERMS cell lines

**Fig. 2:**
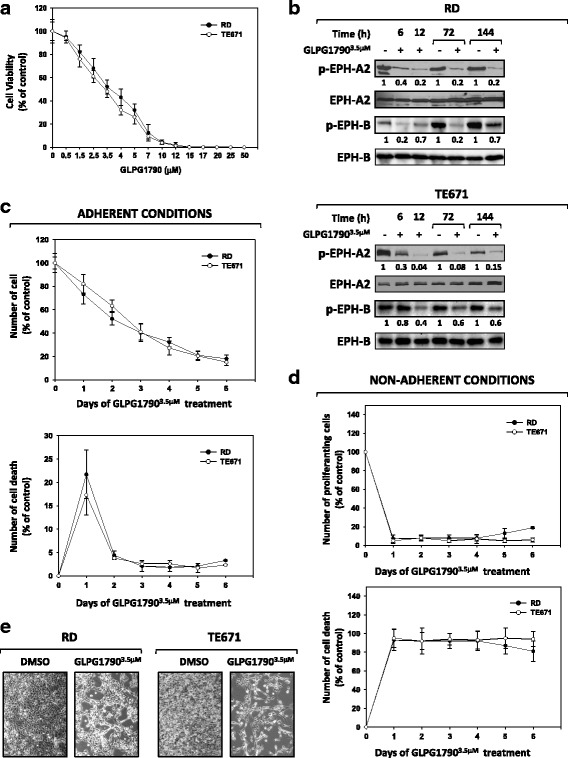
GLPG1790 decreases ERMS cell viability by inducing concomitant cell death and morphology changes in growth arrested cells. **a** Dose-dependent effect of GLPG1790 on viability of RD and TE671 cells after 48 h of treatment. Cell viability was measured by trypan blue dye exclusion test. Results represent the mean values of four independent experiments ±SD. **b** Cell lysates from ERMS cells untreated (DMSO) (−) or treated (+) with 3.5 μM GLPG1790 for the indicated times were analysed by immunoblotting with specific antibodies for indicated proteins. Representative of four independent experiments. **c** RD and TE671 cells, grown in adherent conditions, were treated with 3.5 μM GLPG1790 for the indicated times. Percentage of proliferating (upper panel) or dead (lower panel) cells were obtained by trypan blue dye exclusion test. Results represent the mean values ± SD of four independent experiments. **d** RD and TE671, cells grown in non-adherent conditions, were treated with 3.5 μM GLPG1790 for the indicated times. Percentage of proliferating (upper panel) or dead (lower panel) cells were obtained by trypan blue dye exclusion test. Results represents the mean value of four independent experiments ±SD. **e** Cellular morphology of ERMS cells untreated (DMSO) or treated with 3.5 μM GLPG1790 for 72 h was analysed under light microscope at ×20 magnification. In GLPG1790 treated cells, more elongated cellular bodies were evident, many of which formed multinucleated myotube-like structures. Representative of three independent experiments

The effects of GLPG1790, a pan-EPH receptor kinase inhibitor [21], on RD and TE671 cell viability, were assessed by trypan blue dye exclusion test: cells were treated for 48 h with increasing doses (0–50 μM) of GLPG1790. The drug significantly (*p* < 0.01) decreased ERMS cell viability in a dose-dependent manner, affecting on average the 50% of the cellular viability at a dose of 3.5 μM on both cell lines (Fig. 2a). Immunoblotting experiments showed that in both ERMS cell lines, 3.5 μM GLPG1790 rapidly and persistently decreased the activation/phosphorylation status of EPH receptors, with a stronger action versus EPH-A2 than EPH-B (Fig. 2b). The effects of 3.5 μM GLPG1790 on ERMS proliferation rate and cell viability in adherent or non-adherent (by using Poly-Hema-coated plates) conditions were also investigated. In adherent conditions, GLPG1790 rapidly (1 day) and persistently (1 day to 6 days) inhibited tumour potential growth by 77.9 ± 4.9% on RD (*p* < 0.001) and 71.8 ± 5.1% on TE671 (*p* < 0.001) after 6 days of treatment (Fig. 2c, upper panel). A statistically significant increase of death by 21.8 ± 5.3 in RD (*p* < 0.001) and 17.3 ± 3.6 in TE671 (*p* < 0.001) was detected only 1 day after treatment (Fig. 2c, lower panel). In non-adherent conditions, GLPG1790 treatment drastically reduced the ability of RD and TE671 to grow in suspension (Fig. 2d, upper panel) and resulted in almost total cell death (Fig. 2d, lower panel). Notably, GLPG1790-treated adherent cells exhibited a substantial change in their morphology, with more elongated cellular bodies already at 3 days post-exposure (Fig. 2e). Altogether, these findings indicate that GLPG1790 has a dual action on ERMS population by inducing both growth arrest (cytostatic) and cytotoxic effects, which lead to a muscle-like differentiated phenotype.

### GLPG1790 induces G1 cell cycle growth arrest and reverts ERMS transformed phenotype

**Fig. 3:**
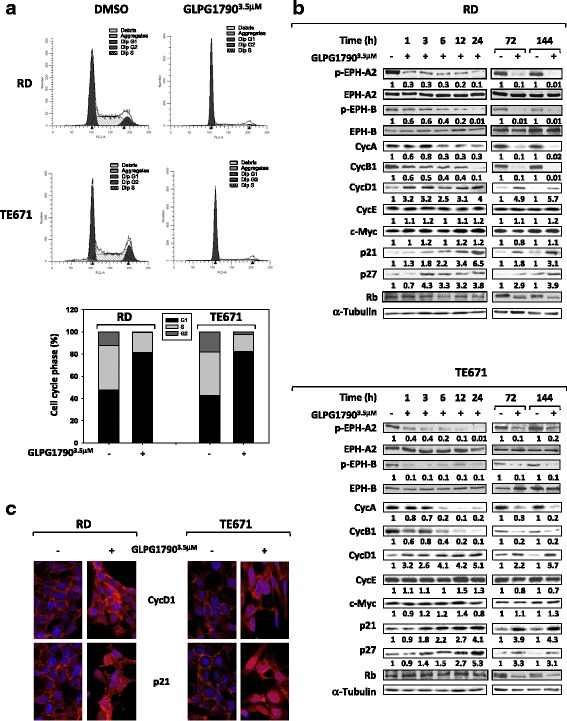
GLPG1790 induces G1 growth arrest and related cell cycle protein modulation. **a** FACS analysis performed on ERMS cells untreated (DMSO) or treated with 3.5 μM GLPG1790 for 24 h. Representative of three different experiments (upper panel). Histograms showing the percentage of cell cycle phases in RD and TE671 cells ± GLPG1790 (lower panel). Results represent the mean value of four independent experiments. **b** Cell lysates from RD and TE671 cells ± GLPG1790 at the indicated times were analysed by immunoblotting with specific antibodies for indicated proteins; α-Tubulin expression shows the loading of samples. Representative of three independent experiments. **c** Immunofluorescence experiments showing the expression and localization of CycD1 and p21 at 72 h after DMSO (−) or GLPG1790 (+) treatment. Representative images captured under ApoTome microscope at 40x magnification

**Fig. 4:**
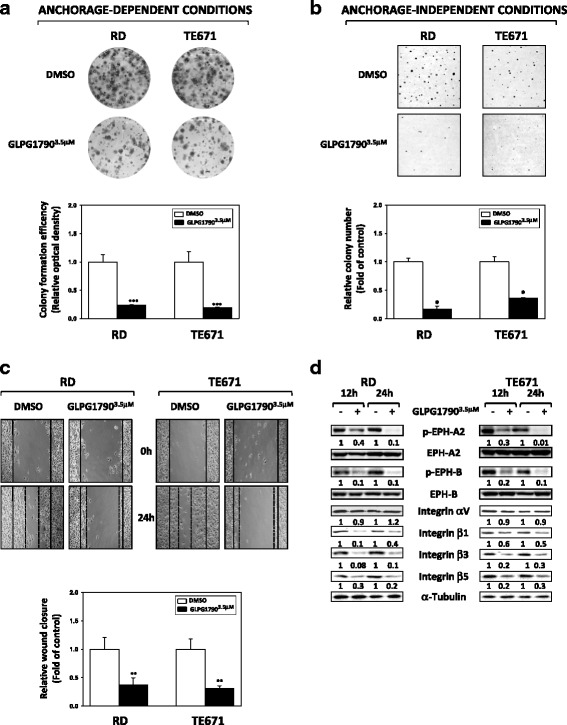
GLPG1790 decreases wound closure and anchorage-dependent or -independent clonogenic ability of ERMS cells. RD and TE671 cells untreated (DMSO) or treated with GLPG1790 were seeded at low concentration in **a** anchorage-dependent conditions or **b** anchorage-independent conditions. Colonies were photographed and counted after 8 days of treatment. Results represent the mean values ± SD of three independent experiments. Statistical significance: *p < 0.05 and ***p < 0.001 compared with the respective control (DMSO), arbitrarily set at 1. **c** Wound healing experiments in RD and TE671 cells. A scratch was made at time 0 and maintained for 24 h in the presence of GLPG1790 or DMSO. The dotted lines represent the edges of the wound. Photographs were taken under light microscope (10x magnification). The migration index was plotted in bar graphs. Statistical significance: **p < 0.01 compared with the respective control (DMSO), arbitrarily set at 1. **d** Cell lysates from RD and TE671 cells treated with or without GLPG1790 for the indicated hours were analysed by immunoblotting with specific antibodies against integrin αV, integrin β1, integrin β3 and integrin β5; α-Tubulin expression shows the loading of samples. Representative of three independent experiments

Cell cycle distribution analysis, performed by flow cytometry on ERMS cells treated for 24 h with 3.5 μM GLPG1790, showed that this drug significantly reduced DNA replication (26.2% ± 2.1 RD and 22.5% ± 3.5 TE671 of S phase) by primarily arresting cells in the G1 phase (79.9% ± 4.7 RD and 86.6% ± 5.1 TE671) of the cell cycle, as reported in Fig. 3a. Consistent with the G1 arrest, GLPG1790 induced an early and persistent decrease of Cyclin A (CycA) and Cyclin B1 (CycB1) expression levels paralleled by the upregulation of p21 and p27 cell cycle inhibitors and by downshift of the retinoblastoma tumour suppressor (Rb) (Fig. 3b), a master regulator of the G1-S transition. Unexpectedly, GLPG1790 rapidly and persistently upregulated the Cyclin D1 (CycD1) protein expression levels whilst no modulation of Cyclin E (CycE) and c-Myc protein expression was observed (Fig. 3b). Immunofluorescence experiments also confirmed that GLPG1790 increases the expression of CycD1 and p21, whose distribution preferentially became nuclear (Fig. 3c). Then, the effects of 3.5 μM GLPG1970 on ERMS oncophenotype were investigated. Eight days after treatment, GLPG1790 reduced the ERMS ability to form colonies in comparison to untreated (DMSO) cells both in anchorage-dependent (78% ± 3.2 for RD and 82.4% ± 0.76 for TE671) and anchorage-independent (83.7% ± 6.1 for RD and 65.2% ± 2.1 for TE671) conditions (Fig. 4a, b, respectively). GLPG1790 also reduced ERMS cell migration as assessed by wound healing assays in which the same fields of confluent cells were pictured immediately after the scratch (time 0 h) and again after 24 h of GLPG1790 preincubation (Fig. 4c, left panel). Drug treatment decreased the level of wound closure to 37% for RD and 31% for TE671 of the control sample (Fig. 4c, right panel). Then, we investigated by Western blot analysis the GLPG1790 effects on the expression levels of integrins, which are involved in cell adhesion and migration. Incubation with 3.5 μM GLPG1790 resulted in a decreased expression of integrin β1, β3 and β5 but not of integrin αV (Fig. 4d). Collectively, these results suggest that the in vitro inhibition of EPH signalling is able to affect ERMS transformed phenotype by reducing cell substrate-dependent or cell substrate-independent proliferation as well as cell migration.

### GLPG1790 promotes myogenic differentiation and affects the in vitro stem cell-like phenotype of ERMS cells

**Fig. 5:**
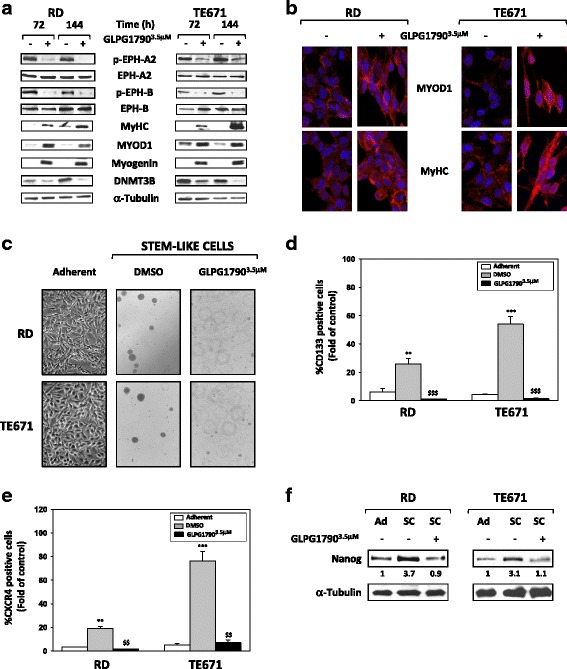
GLPG1790 triggers myogenic differentiation and counteracts ERMS stem-like phenotype. **a** Cell lysates from RD and TE671 cells untreated (DMSO) (−) or treated (+) with GLPG1790 for indicated times were analysed by immunoblotting with specific antibodies for indicated proteins; α-Tubulin expression shows the loading of samples. Representative of three independent experiments. **b** Immunofluorescence experiments showing the expression of MYOD1 and MyHC, at 72 h after GLPG1790 treatment. Images captured under ApoTome microscope at 40× magnification. **c** Representative microphotographs of RD and TE671 cells in adherent conditions (Adherent) and in stem cell (SC) medium after 15 days of incubation in the absence (SC-DMSO) or in the presence of 3.5 μM GLPG1790 (SC-GLPG1790). **d** Histograms of percentage of CD133 positive cells determined by FACS analysis. Results represent the mean values ± SD of four independent experiments. Statistical significance: **p < 0.01, ***p < 0.001 vs. Adherent, ^$$$^ *p* < 0.001 vs. SC-DMSO. **e** Histograms of percentage of CXCR4 positive cells determined by FACS analysis. Results represent the mean value of four independent experiments ± SD. Statistical significance: **p < 0.01, ***p < 0.001 vs. Adherent, ^$$^ *p* < 0.01 vs. SC-DMSO. **f** Western blot analysis of Nanog in protein lysates from RD and TE671 cells in adherent, SC-DMSO or SC-GLPG1790 cultured conditions for 15 days; α-Tubulin expression shows equal loading of samples. Representative of three independent experiments

Since GLPG1790-induced changes in ERMS morphology were suggestive of the acquisition of a myogenic-like phenotype, the expression of specific skeletal muscle markers was evaluated by immunoblotting in ERMS cells treated for 72 and 144 h with GLPG1790 in comparison to untreated control samples. As shown in Fig. 5a, GLPG1790 induced a sustained increase of MYOD1, Myogenin and MyHC protein levels in both RD and TE671 cell lines. Notably, GLPG1790 reduced DNMT3B protein (Fig. 5a), whose expression we have recently shown to restrain ERMS myogenic differentiation [28]. In immunofluorescence experiments, GLPG1790-treated cells displayed a myotube-like morphology with a strong fluorescence signal of MYOD1 and MyHC, this indicating that a proper myogenic differentiation was triggered by the pan-EPH inhibitor (Fig. 5b). The role of the EPH signalling in maintaining the ERMS stem-like population phenotype was also investigated. RD and TE671 cells were cultured in stem cell medium (SC-medium) with or without GLPG1790. Drug treatment drastically reduced the rhabdosphere formation (Fig. 5c) as well as the percentage of CD133 positive cells by 95.6% ± 4.2 in RD and 97.1% ± 6.1 in TE671, compared to the respective SC-DMSO samples (Fig. 5d). A significant decrement in the percentage of CXCR4 positive cells by 91.9% ± 7.7 in RD and 90.6% ± 8.2 in TE671 (Fig. 5e) as well as a downregulation of Nanog protein levels (Fig. 5f) were also evident in the SC-GLPG1790 vs. SC-DMSO comparisons. Taken together, these results suggest that the inhibition of EPH signalling activates a sustained myogenic program in ERMS cells by inducing the sequential expression of myogenic genes and by concomitantly counteracting the ERMS stem-like cell phenotype.

### GLPG1790 radiosensitizes ERMS cells by impairing the DNA double-strand break repair

**Fig. 6:**
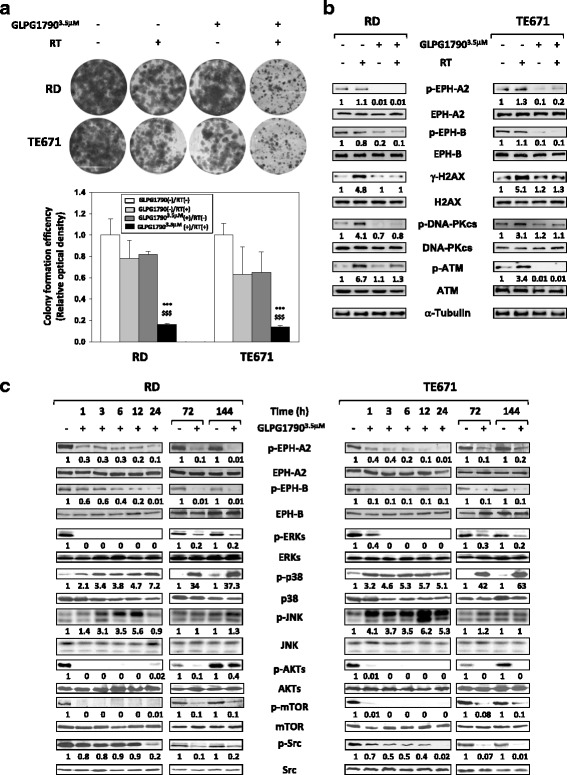
GLPG1790 radiosensitizes ERMS cell lines and modulates signal transduction pathways. RD and TE671 cells untreated or pretreated with GLPG1790 for 24 h were exposed or not to 4 Gy radiation treatment (RT). Two hours after RT, cells were seeded at low concentration for colony assays or lysed for total protein extraction. **a** Representative pictures of colonies stained with crystal violet after 14 days of GLPG(−)/RT(−), GLPG(−)/RT(+) or GLPG(+)/RT(+) treatments (Upper Panel). Colony forming efficiency was calculated by crystal violet absorbance (Lower Panel). Results represent the mean values ± SD of three independent experiments. Statistical significance: ***p < 0.001 vs. GLPG(−)/RT(−), arbitrarily set at 1; ^$$$^ *p* < 0.001 vs. GLPG(−)/RT(+). **b** Cell lysates from RD and TE671 cells were analysed by immunoblotting with specific antibodies for indicated proteins; α-Tubulin expression shows the loading of samples. Representative of three independent experiments. **c** Cell lysates from RD and TE671 treated ± 3.5 μM GLPG1790 for different times were analysed by immunoblotting with specific antibodies for indicated proteins; α-Tubulin expression shows the loading of samples. Representative of three independent experiments

We assessed whether GLPG1790 may sensitize ERMS cells to ionizing radiations by altering DNA damage and/or impairing the molecular mechanisms of DSB (double-strand break) repair. For this purpose, ERMS cells were pretreated or not with GLPG1790 for 24 h and then irradiated with a single dose of 4 Gy; after radiation treatment, GLPG1790 was washed out and colony formation assays were performed. As shown in Fig. 6a, GLPG1790 pretreatment significantly reduced ERMS ability to form colonies with a 85.9 ± 4.4% inhibition in RD and 84.6 ± 1.1% in TE671. Concentration of γ-H2AX, a biomarker of DNA DSBs, and activation status and/or expression levels of ATM and DNA-PKcs, which govern the DSB repair machinery, were determined in cell lysates obtained after 2 h of a single dose of 4 Gy irradiation ± GLPG1790 (Fig. 6b). In the presence of GLPG1790 pretreatment for 24 h, γ-H2AX expression and phosphorylation levels of ATM at Serine 1981 did not increase with ionizing radiations in both cell lines (Fig. 6b). DNA-PKcs activation status (phosphorylation of Threonine 2609) was counteracted only in RD cells (Fig. 6b). These findings indicate that the inhibition of the EPH signalling in ERMS cells reduces H2AX accumulation and downstream DSB repair network.

### GLPG1790 effects on signal transduction in ERMS cells

In order to correlate the effects of GLPG1790 on the ERMS phenotype with specific biochemical mechanisms, we investigated the activity of this compound on key signal transduction pathways linked to RMS development, muscle differentiation and EPH signalling. To this purpose, ERMS were treated with GLPG1790 at different times and Western blot analysis was performed. As shown in Fig. 6c, GLPG1790 treatment (i) rapidly and persistently inhibited the phosphorylation/activation of ERKs, AKTs, mTOR and Src proteins, (ii) induced the rapid and persistent phosphorylation/activation of p38 in both RD and TE671 cell lines and (iii) was able to activate JNKs transiently in RD (from 3 to 12 h) and persistently in TE671 (from 3 to 72 h) cells.

### Silencing EPH-A2 and/or EPH-B2 reproduces GLPG1790-induced effects in ERMS cells

**Fig. 7:**
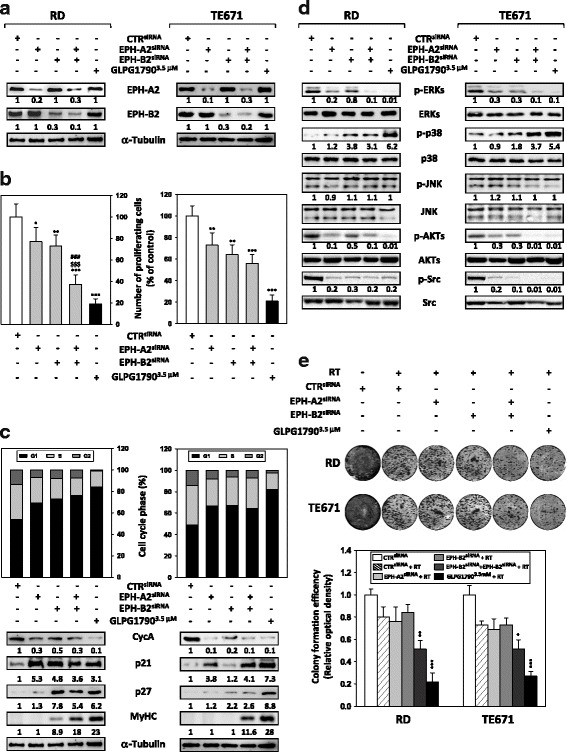
EPH-A2 and EPH-B2 knocking down by RNA interfering affects ERMS cell viability, cell cycle distribution, activation of signal transduction pathways and radiosensitivity. **a** EPH-A2 and EPH-B2 protein expression levels measured by Western blotting at 72 h in RD and TE671 cells after EPH-A2 (EPH-A2^siRNA^) and/or EPH-B2 (EPH-B2^siRNA^) silencing in comparison to samples transfected with non-targeting control siRNA (CTR^siRNA^), arbitrarily set at 1. Images show representative Western blots of three independent experiments; α-Tubulin was used as loading control. **b** Viability of RD and TE671 cells 72 h post-transfection with EPH-A2 and/or EPH-B2 siRNAs was calculated with respect to control cells (CTR^siRNA^) by using trypan blue exclusion staining. Results represent the mean value of three independent experiments ± SD. Statistical significance: *p < 0.05, **p < 0.01, ***p < 0.001 vs CTR^siRNA^, ^$$$^ *p* < 0.001 vs EPH-A2^siRNA^, ^###^ *p* < 0.001 vs EPH-B2^siRNA^. **c** FACS analysis performed on ERMS cells silenced with EPH-A2, EPH-B2 or CTR siRNAs. Histograms show the distribution of cell populations in each phase of the cell cycle. Results represent the mean values of three independent experiments (upper panel). Cell lysates were analysed by immunoblotting with specific antibodies for indicated proteins; α-Tubulin expression shows the loading of samples. Representative of three independent experiments (lower panel). **d** Cell lysates from RD and TE671 cells were analysed by immunoblotting with specific antibodies for indicated proteins; α-Tubulin was used as loading control. Representative of three independent experiments. **e** Representative pictures of colonies stained with crystal violet at 14 days after irradiation (RT) of ERMS cells transfected with EPH-A2^siRNA^, EPH-B2^siRNA^, CTR^siRNA^ or treated with 3.5 μM GLPG1790 (upper panel). Colony formation efficiency was calculated by crystal violet absorbance. Results represent the mean values ± SD of three independent experiments. Statistical significance: *p < 0.05, **p < 0.01, ***p < 0.001 vs. CTR^siRNA^ arbitrarily set at 1 (lower panel)

To investigate if the GLPG1790-mediated effects in RD and TE671 cells were due to the suppression of EPH-A2 and/or EPH-B2 activity, we used specific small interfering RNAs (siRNAs) directed against the EPH-A2 or EPH-B2 subtypes, which share the highest biochemical selectivity profile versus GLPG1790 [21]. A sequence against the *C. elegans* (CTR^siRNA^) was used as a negative control. Western blotting analysis at 72 h after transfection revealed that EPH-A2 protein levels were specifically reduced in EPH-A2^siRNA^-transfected cells (Fig. 7a), whilst EPH-B2 knockdown was obtained only in EPH-B2^siRNA^-transfected samples (Fig. 7a). A significant reduction of both proteins was observed in EPH-A2^siRNA^/EPH-B2^siRNA^ cells compared to those transfected with the negative control siRNA (CTR^siRNA^) (Fig. 7a). GLPG1790 did not perturbate total levels of both EPH-A2 and EPH-B2 proteins (Fig. 7a). At 72 h subsequent to transfection, direct counting for living cells using trypan blue dye exclusion test confirmed that EPH-A2, EPH-B2 and EPH-A2 + EPH-B2 depletion could significantly inhibit the proliferation potential of ERMS cells compared to CTR^siRNA^ cells (Fig. 7b). EPH-A2 silencing inhibited proliferation by 22% in RD and 24% in TE617 cells, EPH-B2 silencing by 24% in RD and 36% in TE671 whilst knocking down of both EPH-A2 and EPH-B2 was able to reduce cell number by 63% in RD and 44% in TE617 cells (Fig. 7b). To further determine whether the reduced ERMS cell growth was due to alterations in cell cycle progression, flow cytometry analysis was performed. Based on PI staining of cellular DNA content, EPH-A2 or EPH-B2 downregulation resulted in a significant GLPG1790-like increase of cell percentage in G1 phase with a concomitant decrease of cell percentage in S and G2 phases (Silencing EPH-A2-RD; G1 69.32 ± 1.9%, S 23.47 ± 2.4%, G2 7.2 ± 0.32%, Silencing EPH-B2-RD; G1 73.13 ± 3.6%, S 18.66 ± 1.5%, G2 8.2 ± 0.29%, Silencing EPH-A2-TE671; G1 66.54 ± 2.8%, S25.25 ± 1.5%, G2 8.2 ± 0.27%, Silencing EPH-B2-TE671; G1 67.32 ± 2.3%, S 26.45 ± 2.3%, G2 6.23 ± 0.17%), whilst CTR^siRNA^-transfected cells rapidly divided and progressed through the cell cycle at high rates (RD G1 52.81 ± 2.5%, S 32.75 ± 2.3%, G2 13.44 ± 1.1%, TE671 G1 49.21.81 ± 1.4%, S 36.57 ± 2.4%, G2 14.22 ± 1.5%,) (Fig. 7c, upper panel). The concomitant silencing of EPH-A2 and EPH-B2 produced the most prominent effects (Silencing EPH-A2 + EPH-B2-RD G1 76.0 ± 3.2%, S 16.21 ± 2.2%, G2 7.47 ± 0.31%, Silencing EPH-A2 + EPH-B2-TE671 G1 64.21 ± 7.8%, S 28.58 ± 4.5%, G2 7.12 ± 0.42%) although the percentage of cells in the different phases did not completely matched with the number obtained by 3.5 μM GLPG1790 treatment (Fig. 7c, upper panel). Consistent with G1 arrest, the expression of different cell cycle regulators was modulated in a GLPG1790-related manner. As GLPG1790, the expression of Cyclin A was significantly downregulated by EPH-A2^siRNA^, EPH-B2 ^siRNA^ or EPH-A2^siRNA^/EPH-B2^siRNA^ in both the cell lines (Fig. 7c, lower panel). As GLPG1790, the expression of the cell cycle inhibitor p21 was increased by EPH-A2 and EPH-A2/EPH-B2 silencing in both cell lines, whilst EPH-B2 had effect only in RD but not in TE671 cells (Fig. 7c, lower panel). The expression of the cell cycle inhibitor p27 was significantly increased by EPH-B2 and EPH-A2/EPH-B2 knocking down mainly in TE671 cells (Fig. 7c, lower panel). Heavy chain of sarcomeric myosin (MyHC) was increased in a GLPG1790-like manner only in the presence of EPH-A2 and EPH-B2 siRNA double transfection in both cell lines (Fig. 7c, lower panel). The effects of EPH-A2^siRNA^, EPH-B2 ^siRNA^ or EPH-A2^siRNA^/EPH-B2^siRNA^ transfection in ERMS oncogenic signalling were also investigated (Fig. 7d). In a GLPG1790-like manner, (i) silencing of EPH-A2 and EPH-A2/EPH-B2 affected ERK phosphorylation/activation in both RD and TE671 cell lines, whilst EPH-B2^siRNA^ reduced ERK activity only in TE671(Fig. 7d); (ii) silencing of EPH-B2 and EPH-A2/EPH-B2 upregulated p38 phosphorylation/activation in both ERMS cell lines, whilst no effects on p38 phosphorylation/activation were observed by EPH-A2 silencing (Fig. 7d); (iii) phosphorylation/activation status of JNKs was not affected by EPH-B2 and/or EPH-B2 knocking down; (iv) transient depletion of EPH-A2, EPH-B2 and EPH-A2/EPH-B2 expression was able to downregulate AKT and Src phosphorylation in both RD and TE671 cells (Fig. 7d). Concerning the possible role of EPH-A2 and/or EPH-B2 in radiosensitizing ERMS cells, no significant effect was observed in EPH-A2^siRNA^ or EPH-B2 ^siRNA^ ERMS cells treated with 4 Gy of RT (Fig. 7e), whilst silencing of both EPH-A2 and EPH-B2 radiosensitized ERMs cells but at a lesser extent than GLPG1790 exposure (Fig. 7e). Taken together, the cellular and molecular effects achieved by using EPH-A2 and EPH-B2 siRNA combined knockdown are comparable to GLPG1790 effects.

### GLPG1790 inhibits tumour growth and radiosensitizes ERMS in xenograft mouse models

**Fig. 8:**
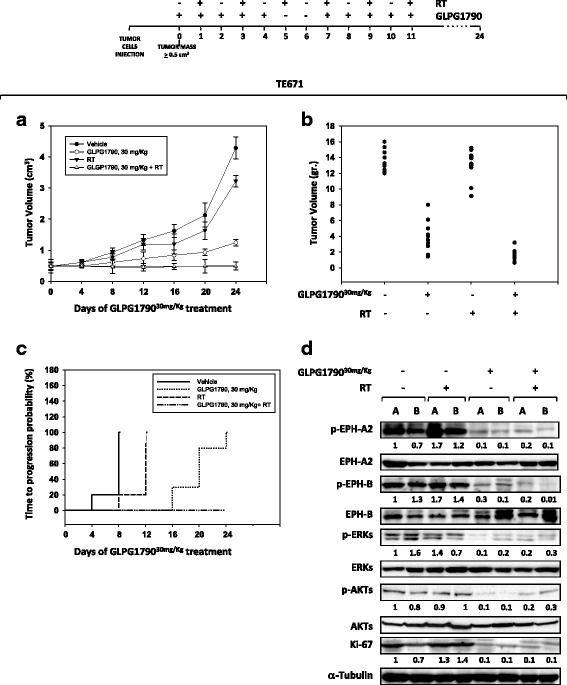
Effects of GLPG1790 combined or not with irradiation on in vivo tumour growth. **a** Growth curve of tumour volumes from xenografted TE671 cell lines, untreated (vehicle), GLPG1790-treated (GLPG1790), irradiated (RT), GLPG1790-pretreated and irradiated (RT + GLPG1790). Tumour volumes were evaluated as describes in methods and represent the mean ± SEM of 10 mice. The upper panel shows the sequential treatments of xenografted mice started when tumours reached a volume of approximate 0.5 cm^3^. GLPG1790 (30 mg/kg) was administered 5 days a week for 2 weeks and before each irradiation, administered on alternate days. **b** Tumour weights in mice untreated or treated with GLPG1790, radiotherapy or combined treatment. **c** Kaplan-Meier estimates for rates of progression for untreated (vehicle), GLPG1790, RT, or GLPG1790 + RT combination in TE671-derived tumours. **d** Phosphorylation/activation status of EPH-A2, EPH-B, ERKs, AKTs and levels of Ki-67 protein in tumours from vehicle, RT, GLPG1790, or GLPG1790 + RT-treated mice (**a**, **b**). Representative Western blot experiment of the 10 tumours analysed. Total EPH-A2, EPH-B, ERKs, AKTs and α-Tubulin immunoblotting were used as loading control (lower panel)

For in vivo experiments, TE671 cell line was chosen due to its intrinsic radioresistance in comparisons with the other tumour cells [30]. When tumour volume reached 0.5–1.0 cm^3^ (T0), GLPG1790 (30 mg/kg) or vehicle (0.5% methylcellulose) were administered by oral gavage 5 days a week for 2 weeks. RT treatment (2 Gy) was performed after the administration of GLPG1790, on alternate days, for 2 weeks and for a total of six applications. Tumour volumes were measured every 4 days for a period of 24 days in untreated (vehicle), GLPG1790-treated (GLPG1790), irradiated (RT) and GLPG1790/irradiated (GLPG1790 + RT) tumours (Fig. 8a). The rate of tumour growth was found to be markedly reduced by GLPG1790 treatment, with a 71% reduction in tumour growth being observed at the end of treatment (*P* < 0.001; Fig. 8a). Furthermore, GLPG1790 + RT combined treatment decreased growth by 83% versus RT alone at end point (Fig. 8a). Tumour weights in mice treated with GLPG1790 decreased significantly ranging from 60 to 80% in GLPG1790(+)/RT(−) and from 80 to 90% in GLPG1790(+)/RT(+) in comparison to controls (Fig. 8b). The number of mice with tumour progression significantly differed across the groups, and this was confirmed by the median values of TTP (Fig. 8c). In the vehicle group, tumour progression occurred within 8 days after the beginning of treatment whilst in the RT group, tumour progression occurred within 12 days after the beginning of irradiation treatment. The treatment with GLPG1790 significantly improved the TTP compared to vehicle (*p* < 0.0001) or RT (*p* < 0.0001). In the GLPG1790 group, tumour progression occurred from the 16th day after the beginning of treatment and completed within the day 24. The most evident improvement was documented when GLPG1790 was coupled with RT: in this group, no tumour progression occurred after the beginning of treatments. Immunoblotting on excised tumours showed that GLPG1790 treatment, combined or not with RT, downregulated EPH-A2, EPH-B, ERK and AKT phosphorylation and reduced Ki-67 expression compared with tumours from vehicle-treated mice (Fig. 8d). Thus, xenografted human TE671-derived tumours are still sensitive to GLPG1790 after 13 days of treatment (Fig. 8d).

## Discussion

**Fig. 9:**
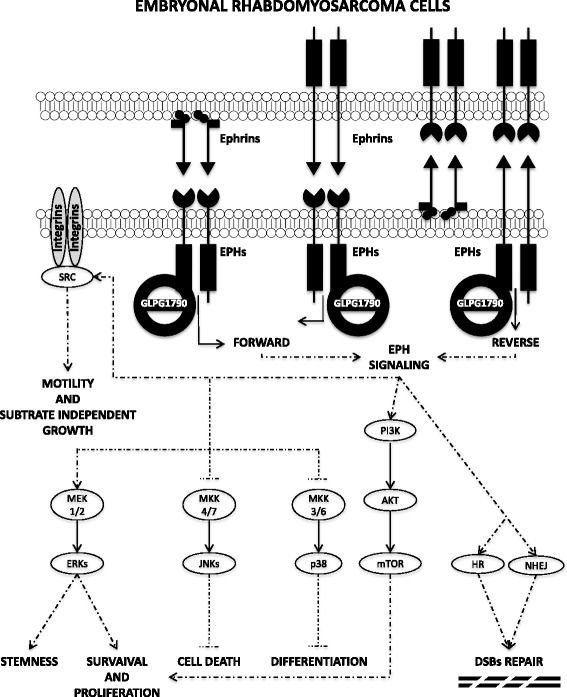
GLPG1790 molecular working models. GLPG1790 inhibits EPH receptor activity and blocks both forward and revers Ephrin signals which, synergistically or individually, support (i) activation of AKT/mTOR and MEK/ERK signalling that support proliferation and cancer stem cell phenotype; (ii) inhibition of pro-apoptotic signal mediated by JNKs; (iii) pro-differentiating signal sustained by p38; (iv) activation of damaged DNA repair molecular mechanisms; (v) motility and invasion abilities by sustaining the SRC-mediated integrin signals

GLPG1790 is a selective and potent pan-inhibitor of the EPH receptors [21], which are overexpressed in many malignancies [12–16] and which are often associated with a poor clinical outcome [13] as well as with a resistance to chemo- [31, 32] and radio-therapy [33–35]. The present study has investigated, for the first time, the possible in vitro antitumour activity of GLPG1790 in ERMS by dissecting the drug-mediated biological effects and molecular mechanisms in RD and TE671 human ERMS cell lines. GLPG1790 was able to decrease the phosphorylation/activation levels of EPH-A2 and EPH-B receptors, which are highly expressed and abnormally activated in ERMS cells lines and tumour biopsies (Fig. 1 for EPH-A2 and [18] for EPH-B). The in vitro inhibition of EPH signalling seems to be a crucial step in reverting ERMS cancer phenotype towards skeletal muscle differentiation, by restricting the expression of proliferative markers and by upregulating the expression of myogenic differentiation markers. Indeed, GLPG1790 treatment at low concentrations (≤ 3.5 μM) induced a significant decrease in cell proliferation and viability, primarily associated with cell cycle arrest, this supporting a cytostatic activity of the drug. Accumulation at G1 phase occurred through several molecular mechanisms, including a significant reduction of both Cyclin A and Cyclin B1 levels, and a marked overexpression of p21 and p27 cell cycle regulators. Furthermore, GLPG1790 exposure led to the downregulation of Rb tumour suppressor, this impairing the transcriptional expression of proliferative genes. The incubation of RD and TE671 cells with GLPG1790 also promoted dramatic morphological changes, with the appearance of more elongated and fused cellular bodies that was consistent with the induction of the myogenic program. In line with this observation, GLPG1790 induced a significant up-regulation of MYOD1 and myogenin, followed by increased levels of MyHC protein, a marker of terminal myogenic differentiation, in both ERMS cell lines. The evidence that impairment of EPH-A2 is linked to myogenesis is in accordance with a report describing the role of EPH/Ephrin interactions in regulating the myogenic program at the expense of self-renewal [36, 37]. Contrarily to what expected, Cyclin D1 levels were rapidly and persistently increased by GLPG1790 treatment in comparison to mocked control cells. The upregulation and perinuclear accumulation of Cyclin D1, confirmed by the immunofluorescence experiments, appear to be an intriguing finding. Indeed, subcellular localization of Cyclin D1 outside the nucleus has been reported to correlate with a lower proliferative index in different cancer types [38, 39], this suggesting that the restriction of Cyclin D1 to the perinuclear region may allow the suppression of G1-S progression. On the other hand, an early accumulation of both Cyclin D1 and D2 is required for the IGF1-mediated myoblast differentiation [40], this supporting a role of specific Cyclin D proteins in the cellular context of muscle terminal differentiation. So, further experiments will be needed to better understand the specific role of Cyclin D1 in the GLPG1790-induced ERMS myogenic differentiation. The biological mechanisms altered by GLPG1790 include the modulation of several signal transduction pathways, which are involved in promoting oncogenic transformation and progression in many types of tumours, and which have been previously linked to EPH RTKs [6–8]. In particular, in both ERMS cell lines, GLPG1790 concomitantly reduced activation status of AKT, mTOR, ERK, JNK and Src proteins, whose related signalling are known to promote ERMS development and to block terminal muscle differentiation [22, 23, 41–44]. Notably, p38 phosphorylation levels were dramatically increased by GLPG1790 treatment, confirming the key role of this mitogen-activated protein kinase in the reactivation of the terminal differentiation program in both RD and TE671 cells [22, 28, 41–44]. Our data also suggest that the GLPG1790-mediated acquisition of the myogenic-like phenotype and the enhanced expression of specific myogenic genes, such as MYOD1, myogenin and MyHC, seems to pass through the reduction of DNMT3B protein levels, whose knockdown has recently been shown to be a key event in reactivating the ERMS terminal differentiation program [28]. According to our recent report that shows how the MEK/ERK pathway plays a prominent role in maintaining the stem-like phenotype of ERMS cells [22], we found that GLPG1790 dramatically prevented rhabdosphere formation and downregulated the expression of the stem cell markers CD133, CXCR4 and Nanog. The finding that GLPG1790 treatment induces cell cycle alteration and commits ERMS cells to myogenesis suggests that this compound has a therapeutic potential as a differentiating agent in ERMS tumours. Differentiation therapy has been shown to have a significant clinical antitumour activity in acute promyelocytic leukemia, and promising preclinical activity in liposarcoma and osteosarcoma [45, 46], this outlining the importance to test the antitumour effects of this EPH inhibitor in xenograft models [21]. We also found that GLPG1790 exposure was able to significantly reduce the migratory as well as the clonogenic capacity of RD and TE671 cells by altering the expression of specific proteins, including members of the integrin superfamily, this supporting the role of EPH signalling in regulating the migratory behaviour and metastatic potential of cancer cells [6–8]. Combined siRNA knockdown of both EPH-A2 and EPH-B2 genes, herein, shown and known [18] to be overexpressed in ERMS tumours, replicated many of the phenotypic effects observed in ERMS cells after drug exposure, confirming that GLPG1790 activity is mediated by the efficient impairment of EPH activity [21]. Finally, our data underline the role of GLPG1790 exposure in potentiating the effects of radiotherapy, which usually works by inducing DSBs as well as by inhibiting the non-homologous end joining (NHEJ) and the homologous recombination (HR) DNA repair pathways in exposed tumour cells [47]. Indeed, GLPG1790 enhanced radiosensitivity of ERMS cell lines, as demonstrated by the clonogenic survival reduction of more than 90%, altering the accumulation of DNA DSBs, as confirmed by the impaired expression of the phosphorylated form of the H2AX histone [48]. Even if the relationship between EPH/Ephrin signalling, DSB repair machinery and response to RT is still largely unknown; a correlation between EPH overexpression and the acquisition of a radioresistant phenotype has been reported in other solid tumours [34, 35, 49, 50]. Here, we showed that GLPG1790 abrogates the RT-induced ATM and DNA-PKcs phosphorylation, whose activation is linked to the HR and NHEJ pathways, respectively [47]. Augmentation of radiation response by GLPG1790 treatment was also confirmed by our preliminary in vivo experiments, in which the combination therapy with GLPG1790 and fractionated radiation was significantly more effective than the drug or the RT alone in reducing tumour masses. The current treatment for patients with RMS is a combination of surgery, chemotherapy and/or radiotherapy. However, the development of resistance to chemotherapy and radiotherapy is often a significant limiting factor, leading to therapeutic failures and poor survival [5]. Since EPH signalling seems to sustain the oncogenic and radioresistant phenotype of ERMS by regulating several molecular mechanisms [50] as schematized in Fig. 9, the combined use of GLPG1790 and RT may represent an attractive strategy to make clinical treatment of ERMS tumours more effective. Further studies in ERMS animal models will be necessary to assess if GLPG1790 has a significant activity in preventing the in vivo radioresistance. Furthermore, since chemical inhibition of the DNA repair machinery has been proposed as a novel strategy for cancer treatment, radiosensitizing effects conferred by GLPG1790 in ERMS cells may also open a new field of promising approaches in the treatment of other cancer types that over-express EPH family members.

## Conclusions

The results of this study demonstrate the preclinical in vitro and in vivo antitumour activity of GLPG1790, a new potent pan-inhibitor of EPH receptors, in human ERMS cells. In particular, GLPG1790 induces G1-growth arrest and commits ERMS cells towards skeletal muscle differentiation. Drug treatment prevents rhabdosphere formation and downregulates stem cell markers. GLPG1790 also radiosensitizes ERMS cells by impairing the DNA double-strand break repair pathway. Finally, RMS xenografts exhibited greater sensitivity to the combined GLPG1790 and radiotherapy treatment.

## Abbreviations

ATM: Ataxia-telangiectasia mutated
DNA-PKcs: DNA-dependent protein kinase
EPH: Erythropoietin-producing hepatocellular
MyHC: Myosin heavy chain
MYOD1: Myoblast determination protein 1
